# Relationship Between Scheimpflug-Based Ocular Biomechanics and Myopic Maculopathy

**DOI:** 10.3390/bioengineering13060658

**Published:** 2026-06-04

**Authors:** Beatriz Costa Vieira, Diogo Rodrigues, João Heitor, João Coelho, Paulo Sousa, Miguel Lume, Angelina Meireles, Renato Ambrósio, Pedro Menéres, João Melo Beirão, Pedro Manuel Baptista

**Affiliations:** 1 Ophthalmology Department, Unidade Local de Saúde de Santo António, 4099-001 Porto, Portugal; 2 Department of Cornea and Refractive Surgery, Instituto de Olhos Renato Ambrósio, Rio de Janeiro 20520-050, Brazil; 3 Department of Ophthalmology, Federal University of the State of Rio de Janeiro (UNIRIO), Rio de Janeiro 21941-617, Brazil; 4 Department of Ophthalmology, Federal University of São Paulo (UNIFESP), São Paulo 04039-060, Brazil; 5 Instituto de Ciências Abel Salazar, University of Porto, 4050-313 Porto, Portugal

**Keywords:** high myopia, ocular biomechanics, myopic maculopathy

## Abstract

Ocular biomechanics may contribute to the variability of structural and functional outcomes in highly myopic eyes, but their role in myopic maculopathy remains unclear. This retrospective cohort study investigated whether in vivo corneal biomechanical parameters are associated with macular structural phenotypes and longitudinal functional changes. Fifty-four eyes with high myopia (≤−6 D; mean SE −15.6 ± 6.6 D) were evaluated at baseline and after 5.0 ± 0.1 years. Biomechanics were assessed with Corvis Scheimpflug Technology^®^, macular structure was assessed with SD-OCT (6 × 6 mm), and function was assessed with Microperimeter MP-3 (CPS, dB). Foveoschisis was associated with higher A2 deformation amplitude (0.371 vs. 0.333 mm, *p* = 0.014) and SSI (0.986 vs. 0.827, *p* = 0.035). Staphyloma showed changes in the highest concavity radius (7.13 vs. 6.17 mm, *p* = 0.002), A1 deformation amplitude (0.150 vs. 0.132 mm, *p* = 0.001), and maximum deflection amplitude (1.03 vs. 1.19 mm, *p* = 0.013). Softer corneal parameters correlated with less functional loss, while stiffer parameters correlated with greater decline; similar trends were observed for fixation stability. These findings suggest that biomechanical profiles may vary across macular phenotypes and could be associated with functional evolution in highly myopic eyes.

## 1. Introduction

High myopia (HM) is defined as a refractive error of at least −6.00 diopters (D) [1]. When HM is accompanied by choroidal and retinal degeneration with different macular pathological changes, it is referred to as pathologic myopia (PM) [2,3].

Currently, the prevalence of myopia is increasing, and in 2050, nearly 50% of the global population is expected to have myopia, with HM incidence projected to reach 10% [4]. While HM is often stable, progressive maculopathy can severely impact visual function, especially in active, young individuals, often necessitating vitreoretinal surgery. PM-associated macular alterations are a leading cause of serious visual impairment and even blindness not only in East Asian countries such as Japan [5], Singapore [6] and China [7] but even in the United States [8] and Europe [9,10]. Thus, HM represents a significant and growing global health burden that requires urgent attention.

The physiopathological basis for progressive degeneration of the macular region in these eyes was proposed by Panozzo et al. [11] to be the vitreomacular traction forces applied to its anatomically disproportionate structure. The concept of maculopathy associated with vitreomacular traction in HM eyes—“Myopic Traction Maculopathy” (MTM)—has become more defined over time, evolving into a broader term that now encompasses various alterations and mechanisms beyond vitreomacular traction itself, such as posterior staphyloma (PS), myopic foveoschisis (FS), and myopic macular hole (MH) [12]. However, the causal relationships between these typical changes in eyes with PM remain a subject of discussion and study.

The prevalence of MTM is variably reported between 9% and 34% in highly myopic eyes [11]. On the other hand, there is consensus on three key determinants of MTM progression: axial length, anterior traction exerted by the vitreous, and posterior traction exerted by the staphylomatous component [3,13]. Additional factors such as remnants of the vitreous cortex, tangential traction and epiretinal membrane formation are also believed to contribute [13]. However, identifying the predominant factor in each eye and predicting the medium- and long-term risk of progression requires a deeper understanding of the relationship between the applied tensions and their interaction with a given scleral anatomy and biomechanical behavior.

Detailed analyses of abnormal structures at the vitreoretinal and retinochoroidal interfaces achieved with advances in Spectral-Domain Optical Coherence Tomography (SD-OCT) have helped the development of a consistent anatomical classification of MTM by Medrano et al. [3]. Although this classification has demonstrated prognostic value [14], it is primarily based on structural features and does not incorporate functional parameters such as visual acuity or retinal sensitivity. In addition, it does not account for potential differences in ocular biomechanical behavior.

Reports of biomechanical characterization of myopic eyes are increasing in the literature, establishing that eyes with HM have different biomechanics and that there may be no interchangeability between anatomy and the biomechanical behavior of tissues, thereby positioning biomechanical assessment as a potentially distinct and independent prognostic factor [15,16,17,18,19,20,21,22].

Nowadays, the in vivo biomechanical behavior of the eyeball can be measured through the analysis of corneoscleral tissue movement using ultra-high-speed Scheimpflug imaging during non-contact tonometry with the Corvis ST (OCULUS, Germany) [23], including the use of several evolving, validated algorithms as descriptors of ocular biomechanical behavior and its stiffness [24,25,26,27,28].

The main purpose of the present study is to analyze in vivo ocular biomechanics in highly myopic eyes with MTM and to investigate whether baseline biomechanical parameters are associated with macular structural phenotypes and with longitudinal functional progression, as assessed by fixation stability and retinal sensitivity.

## 2. Materials and Methods

### 2.1. Design and Study Population

This retrospective, longitudinal cohort study included eyes with diagnosed high myopia (HM; spherical equivalent ≤ −6.0 diopters [D]) and different forms of myopic traction maculopathy. Patients under regular follow-up at the Surgical Retina Department of Centro Hospitalar e Universitário do Porto (Unidade Local de Saúde Santo António) between January and July 2020 were identified through clinical records. Standardized ophthalmologic examinations performed during routine clinical follow-up at baseline and at approximately 5-year follow-up (January to July 2025) were retrospectively reviewed and analyzed.

Exclusion criteria were: previous intraocular surgery, previous corneal ablative procedures, presence of corneal dystrophies or other corneal or scleral diseases, pterygium or other conjunctival conditions, inability to fixate, phthisis bulbi or other ocular decompensated status, and cognitive inability to perform the exams.

**Figure 1:**
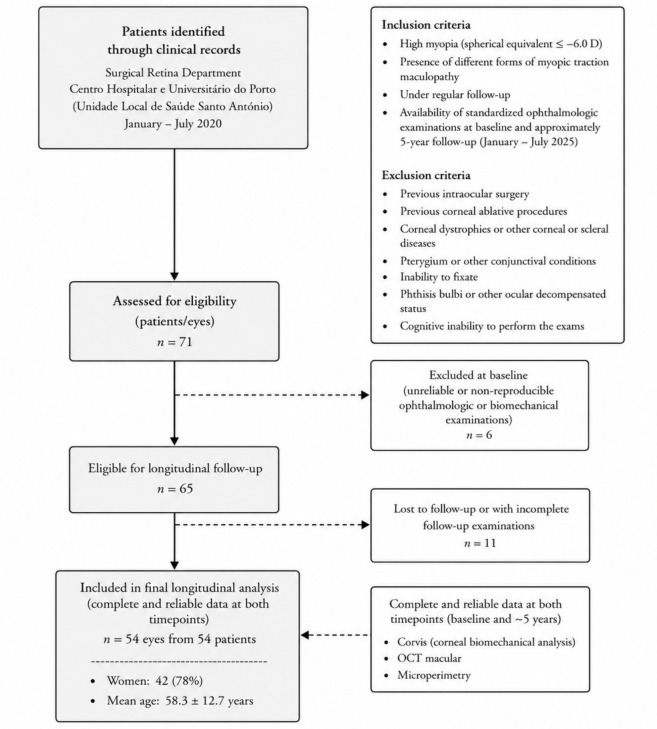
Flowchart of patient selection and longitudinal follow-up. Abbreviations D, diopters; OCT, optical coherence tomography.

Eyes with unreliable or non-reproducible ophthalmologic or biomechanical examinations at baseline were excluded. Additionally, participants lost to follow-up or with incomplete follow-up examinations were excluded from the final longitudinal analysis. Only eyes with complete and reliable data available at both study time points were included in the final analysis. The patient selection process and reasons for exclusion are summarized in Figure 1.

The study adhered to the tenets of the Declaration of Helsinki. Ethical approval was obtained in October 2021 from the Departamento de Ensino, Formação e Investigação (DEFI) of the Centro Hospitalar e Universitário do Porto (No. 130-DEFI-132-CE). The requirement for informed consent was waived by the Ethics Committee due to the retrospective use of fully anonymized and confidential clinical data, with no identifiable individual information included in the analysis.

### 2.2. Demographic and Clinical Data

All patients underwent a thorough ophthalmological evaluation, including slit-lamp and indirect mydriatic fundoscopic exams. We objectively analyzed the lens status, subjective refraction and spherical equivalent (SE). Demographic data were collected—namely, gender and age.

### 2.3. Ocular Biomechanical and Biometric Data

Biomechanical assessment was performed with Corvis Scheimpflung Technology^®^ (Oculus, Wetzlar, Germany). Only exams with an ‘OK’ quality score were included. Parameters from the three significant time points were recorded: time from the initiation of air puff until the first applanation (A1), second applanation (A2) and highest concavity (HC). Additional first-generation parameters from the maximum deformation on the oscillatory phase (Max) and from Whole Eye Movement (WEM) were analyzed. The second-generation parameters included the Corvis Biomechanical Index (CBI), the Stiffness Parameter in A1 (SP-A1) and the Stress–Strain Index (SSI). All Scheimpflug-based parameters used in the study and their explanations are summarized in Table 1.

### 2.4. Characterization of Macular Structure

The macular structure was assessed at baseline by spectral-domain optical coherence tomography (SD-OCT; Heidelberg^®^ Engineering, Heidelberg, Germany). Only exams with a good quality of acquisition were analyzed, and we collected automated data on the central foveal thickness (CFT, central 1 mm circle) and manually measured subfoveal choroidal thickness (CT) without an enhanced depth-image (EDI) protocol. Manual CT measurements were performed by an ophthalmology specialist experienced in OCT interpretation and retinal imaging analysis. Measurements were obtained perpendicularly from the outer border of the retinal pigment epithelium to the choroid–sclera interface at the foveal center. Within the 6 × 6 mm square area centered on the fovea, we recorded: the presence of foveoschisis (T1, T2, and T3) and areas of external retinal atrophy or fibrosis (A2, A3, and A4) according to the proposed grading system for myopic maculopathy 2, the presence of vitreomacular traction (VMT) according to the International Vitreomacular Traction Study Group classification29; and the presence of posterior pole staphyloma [29]. Eyes presenting with a myopic foveal macular hole in SD-OCT (T4 and T5)2 were excluded.

### 2.5. Macular Microperimetry Assessment

The study of macular function was carried out at baseline and at the end of the follow-up with an MP-3 microperimeter (NIDEK^®^, Aichi, Japan). The exams were performed against a 31.4 asb white background, with a Goldmann V white stimulus (34 dB dynamic range) through a fast (4–2) strategy and a single-cross red (2°) fixation target. We only analyzed reliable exams. Data were collected on the mean retinal sensitivity of the 24 measured points in the foveally centered 12° polygon (CPS) and on the fixation capacity of the eye through the analysis of the fixation stability (% of fixations within the 2° (F2°) and 4° (F4°) foveally centered circles), as well as on the bivariate contour ellipse areas (BCEA) of the 1 (68.2%), 2 (95.4%), and 3 (99.6%) standard deviations (SD) of the total fixations (BCEA 1SD, 2SD, and 3SD, respectively). Deltas of variation for each variable were calculated as the difference between the value at the end of follow-up and the value at baseline (ΔF2, ΔF4°, ΔBCEA_1SD, ΔBCEA_2SD, ΔBCEA_3SD, and ΔCPS).

### 2.6. Study Steps

The principal analyses focused on longitudinal changes in microperimetry-derived functional parameters over follow-up and on the associations between these functional changes and baseline corneal biomechanical parameters. Secondary analyses evaluated differences in baseline biomechanical characteristics across distinct myopic macular structural phenotypes. To address these objectives, the following analyses were performed:

1. General analysis (baseline characterization): anatomical, biomechanical, and functional characterization of the study cohort at baseline was performed.
2. Four independent comparative analyses were conducted to assess differences in baseline ocular biomechanical parameters according to macular structural status. Specifically, comparisons were performed between:
  - Eyes with retinal splitting/foveoschisis (foveoschisis group, *n* = 19) and those without this alteration;
  - Eyes with fibrotic and/or atrophic changes of the outer retina (atrophy/fibrosis group, *n* = 33) and those without this alteration;
  - Eyes with anterior vitreomacular traction (VMT group, *n* = 9) and those without this alteration;
  - Eyes with posterior pole staphyloma (staphyloma group, *n* = 31) and those without this alteration.
3. Correlation analysis: Univariable correlation analyses were performed to investigate the correlations between baseline ocular biomechanical parameters and longitudinal changes in macular microperimetric functional outcomes over the follow-up period, considering the entire study cohort.
4. Additionally, multivariable linear regression models were developed to identify independent associations between baseline biomechanical parameters and ΔCPS 12°. Candidate predictors included baseline biomechanical parameters that showed significant or near-significant associations in univariable analyses (*p* < 0.10) and/or were considered clinically relevant. A stepwise selection procedure was applied (entry *p* < 0.05, removal *p* > 0.10) to derive the final models. To reduce the risk of multicollinearity, correlations between predictors were assessed, and variance inflation factors (VIF) were calculated, with a predefined threshold of VIF > 5 indicating collinearity. In such cases, only one of the correlated variables was retained based on clinical relevance. Given the limited sample size (*n* = 54), the number of predictors included in each final model was restricted to maintain an appropriate ratio between sample size and model complexity. Potential confounding by baseline age, SE, CFT and CT was assessed.

### 2.7. Statistical Analysis

Statistical analysis was performed with the IBM SPSS statistical software package, version 24.0 (SPSS^®^, Chicago, IL, USA). Normality of the data was confirmed by the Shapiro–Wilk test. Levene’s test was used to look for homogeneity of variances, and Student’s *t*-test was used to compare variables between groups. When nonparametric tests were needed, the Wilcoxon rank-sum test was applied. Possible correlations were studied with Pearson’s correlation coefficient. Values are shown as mean ± standard deviation unless otherwise specified. All *p*-values (*p*) were 2-sided, and *p*-values < 0.05 were considered significant.

## 3. Results

Initially, 71 patients were recruited for the study. Of these, 6 were excluded due to unreliable or non-reproducible examinations at baseline, and 11 did not complete the follow-up evaluation. Therefore, the final analysis included 54 eyes from 54 patients, of whom 42 (78%) were women, with a mean age of 58.3 ± 12.7 years (Figure 1). Baseline characterization of the study sample, including with respect to microperimetric data and information on macular structure-the presence of foveoschisis, atrophy/fibrosis, vitreomacular traction (VMT), and posterior pole staphyloma, which were used to define the MTM subgroups, is summarized in Table 2 for the overall study population and stratified by MTM subgroup.

### 3.1. Subgroup Analysis

Subgroup analyses comparing baseline ocular biomechanical parameters according to macular structural status are summarized in Table 3a,b. Abbreviations are defined in Table 1.

Eyes with foveoschisis exhibited higher A2 deformation amp. and A2 deflection length (0.371 ± 0.049 vs. 0.333 ± 0.044 mm (*p* = 0.014) and 3.392 ± 1.076 vs. 2.631 ± 0.626 mm (*p* = 0.007), respectively). In addition, eyes with foveoschisis showed higher SSI values (0.986 ± 0.281 vs. 0.827 ± 0.196, *p* = 0.035).

Eyes with outer retinal atrophy and/or fibrosis exhibited lower HC Time (17.132 ± 0.723 vs. 17.498 ± 0.354 ms, *p* = 0.032).

No significant differences in baseline biomechanical parameters were detected between eyes with and without vitreomacular traction.

**Figure 2:**
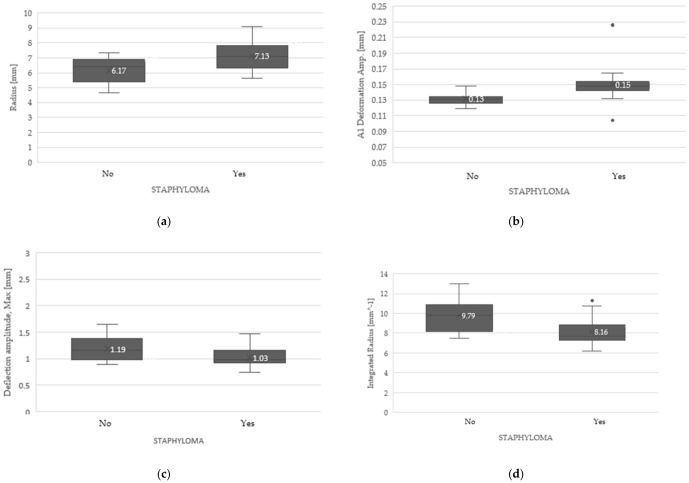
Boxplots comparing corneal biomechanical parameters between highly myopic eyes with and without posterior staphyloma. (**a**) Radius [mm]; (**b**) A1 deformation amplitude [mm]; (**c**) maximum deformation amplitude [mm]; (**d**) integrated radius [mm^−1^]. The numerical values displayed within each box represent the mean value for each variable in the corresponding subgroup. Boxes represent the interquartile range (IQR), the horizontal line within each box indicates the median, and dots(outliers) represent outliers.

In contrast, eyes with posterior pole staphyloma exhibited a higher Radius, A1 deformation amp. and A1 deflection length (7.126 ± 0.896 vs. 6.168 ± 0.850 mm, *p* = 0.002; 0.150 ± 0.019 vs. 0.132 ± 0.008 mm, *p* = 0.001 and 2.369 ± 0.178 vs. 2.225 ± 0.173 mm, *p* = 0.019, respectively) and lower deflection amp. max (1.028 ± 0.175 vs. 1.192 ± 0.235 mm, *p* = 0.013). Additional differences were observed in HC dArc length, max inverse Radius, and integrated radius, which were lower in eyes with staphyloma (−0.152 ± 0.031 vs. −0.076 ± 0.130 mm, *p* = 0.041; 0.175 ± 0.020 mm^−1^ vs. 0.249 ± 0.105, *p* = 0.016; and 8.164 ± 1.428 vs. 9.794 ± 1.646 mm^−1^, *p* = 0.002). Representative boxplots of selected biomechanical parameters are shown in Figure 2.

### 3.2. Correlation Analysys

**Figure 3:**
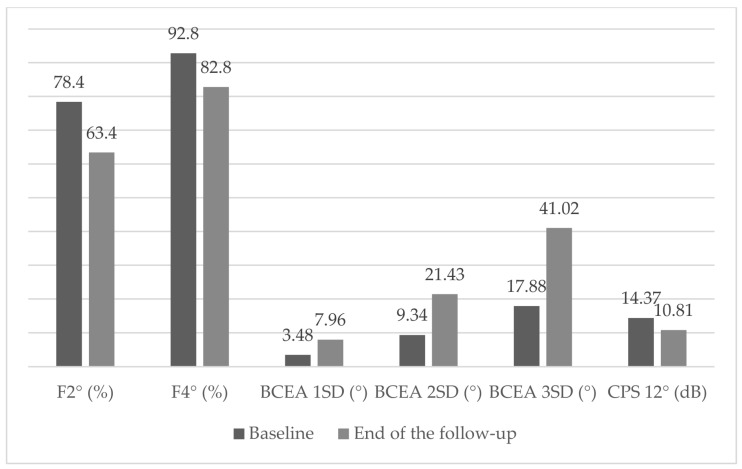
Longitudinal changes in microperimetric functional parameters over 5-year follow-up. Abbreviations: CPS 12, retinal sensitivity in the foveally centered 12° polygon; F2 and F4, proportion of fixations within the 2° and 4° foveally centered circles; BCEA 1SD, 2SD and 3SD, bivariate contour ellipse areas of the 1 (68.2%), 2 (95.4%) and 3 (99.6%) standard deviations of the total fixations; SD, standard deviation.

Results with respect to longitudinal changes in microperimetric parameters and correlation analysis between baseline corneal biomechanical parameters and 5-year functional outcomes are presented in Table 4 (abbreviations are described in Table 1). Overall, longitudinal changes suggested worsening functional performance over time, characterized by negative deltas in F2°, F4°, and CPS and positive deltas in BCEA indices. Given the large number of biomechanical parameters and outcomes tested, correlation analyses should be interpreted as exploratory, and uncorrected *p*-values are reported throughout. A graphical representation of longitudinal changes in microperimetric parameters is shown in Figure 3.

Among the evaluated outcomes, ΔCPS 12° and ΔBCEA indices showed the most frequent associations with baseline biomechanical parameters. Four biomechanical parameters demonstrated consistent correlations across all BCEA measures (1SD, 2SD, and 3SD)—namely, HC dArc length, A2 dArc Length, dArcLengthMax, and max inverse radius, all showing negative correlations with ΔBCEA. In addition, all ΔBCEA measures showed positive correlations with the second-generation biomechanical parameter of SSI.

**Figure 4:**
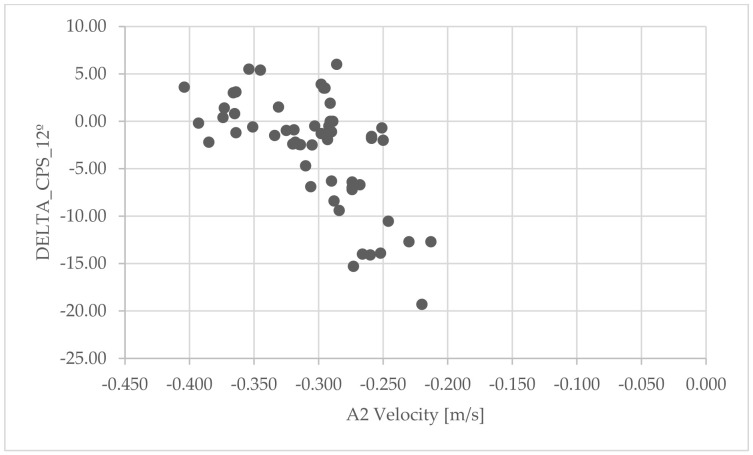
Scatterplot demonstrating the relationship between baseline A2 Velocity [m/s] and the 5-year change in CPS 12° (Δ CPS 12°). Each point represents one eye.

**Figure 5:**
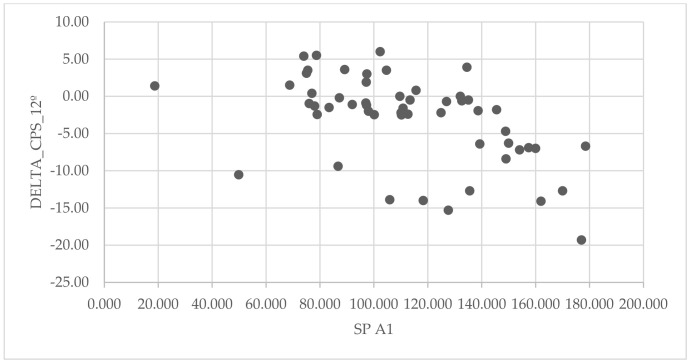
Scatterplot demonstrating the relationship between baseline SP-A1 and the 5-year change in CPS 12° (Δ CPS 12°). Each point represents one eye.

ΔCPS 12° showed multiple correlations with first-generation dynamic corneal response parameters, particularly A2 Velocity and A1 Time, as well as with deformation-related parameters such as def. amp. max, peak dist., and HC deflection length. In addition, ΔCPS showed a negative correlation with the second-generation stiffness parameter, i.e., SP-A1. Representative scatterplots illustrating the correlations between ΔCPS 12° and baseline A2 Velocity and SP-A1 are presented in Figure 4 and Figure 5, respectively.

The remaining correlations, including the fixation stability indices (ΔF2° and ΔF4°), are presented in Table 4.

Linear regression models were used to evaluate the baseline biomechanical parameters independently associated with changes in ΔCPS 12°. Significant independent associations were observed for age (β =0.284; *p* =0.007), SE (β = 0.223; *p* = 0.047), central foveal thickness (β =−0.011; *p* =0.056), choroidal thickness (β = 0.070; *p* = 0.001), A1 velocity [m/s] (β = −239.98; *p* = 0.004), A2 velocity [m/s] (β = −85.29; *p* =0.003) and HC deflection length [mm] (β = 5.80; *p* = 0.029) (R^2^ = 0.686; adjusted R^2^ = 0.564; overall model *p* = 0.001). Results of the multivariable analysis are summarized in Table 5.

## 4. Discussion

The present study was designed to investigate whether corneal biomechanical behavior reflects structural phenotypes and/or could be associated with retinal functional loss over time in myopic eyes.

The cornea’s viscoelastic properties allow it to resist and recover from deformation, reflecting both stromal collagen integrity and overall ocular connective tissue behavior [29,30]. Using the Corvis ST, these properties can be assessed dynamically through multiple parameters describing the corneal response to the air-puff stimulus. Some parameters primarily reflect corneal deformability, including Def. Amp. Max, HC Deflection measures, Peak Dist., and Max InverseRadius, whereas others translate resistance to deformation or stiffness of the corneoscleral unit, such as A1 Time, A2 Velocity, IOP-related metrics, and SP-A1. In addition, composite indices including CBI and SSI summarize overall biomechanical behavior and intrinsic tissue stiffness [15,31]. Detailed definitions of these parameters are provided in Table 1.

Together, these measures provide a window into the characteristics of ocular connective tissue, which can be particularly relevant in highly myopic eyes, as variations in these parameters may underlie the structural and functional differences observed across macular phenotypes [17,32].

This study included 54 highly myopic eyes (mean spherical equivalent of −15.6 ± 6.6 D), stratified into four macular phenotypes: foveoschisis, outer retinal atrophy or fibrosis, vitreomacular traction, and posterior pole staphyloma. This distribution allowed for the analysis of tractional, degenerative, and ectatic manifestations of high myopic maculopathy in a clinically meaningful cohort.

### 4.1. Ocular Biomechanical Profiles Across High-Myopia Subgroups

The comparative analyses identified distinct ocular biomechanical patterns across different macular structural phenotypes, suggesting that corneal dynamic response parameters may be associated with heterogeneous biomechanical behavior in highly myopic eyes. However, given the exploratory nature of these subgroup analyses and the number of comparisons performed, these findings should be interpreted cautiously and considered hypothesis-generating.

Eyes with foveoschisis exhibited significantly greater second applanation deformation amplitude (A2 Deflection Amp.) and second applanation deflection length (A2 Deflection Length), together with higher SSI values. Increased A2 parameters suggest greater posterior corneal excursion during the air-puff response, reflecting increased corneal deformability. In contrast, SSI represents the intrinsic stiffness of the corneoscleral shell and is considered relatively independent of intraocular pressure [33]. The coexistence of increased deformation parameters with higher SSI may therefore reflect differences between local corneal deformability and the global biomechanical behavior of the corneoscleral unit. While the cornea may exhibit greater dynamic deformation, a relatively stiffer scleral structure could limit the ability of the posterior segment to accommodate mechanical forces. The potential relationship between reduced scleral compliance and vitreomacular tractional forces has been proposed in the literature but cannot be confirmed by the present data.

Eyes with outer retinal atrophy and/or fibrosis exhibited lower values of the highest concavity time (HC Time). However, no additional significant differences were observed across most deformation- and stiffness-related parameters. These findings may suggest that degenerative outer retinal changes are less consistently associated with global ocular biomechanical behavior than tractional or ectatic phenotypes, although the limited sample size precludes definitive conclusions.

Baseline biomechanical parameters did not differ significantly between eyes with and without vitreomacular traction. It is noteworthy that this subgroup comprised the smallest number of subjects in our cohort (9 of 54 eyes), which may have limited the statistical power to detect subtle differences. Therefore, the absence of statistically significant differences should be interpreted cautiously, as it may reflect either a true lack of association between VMT and global ocular biomechanical parameters or an insufficient sample size to detect small effects.

Eyes with posterior pole staphyloma exhibited the most pronounced biomechanical differences in our cohort. Conceptually, posterior staphyloma may be interpreted as a structural manifestation of biomechanical fragility of the posterior ocular wall, analogous to an ectatic process of the posterior segment. From this perspective, biomechanical behavior differences would be expected in eyes presenting this phenotype.

The observed pattern of deformation parameters suggests altered energy transmission and dissipation within the ocular wall. During the air-puff response, more compliant corneas tend to dissipate mechanical energy through greater deflection, whereas relatively stiffer corneas may transmit a larger proportion of the applied stress to the posterior ocular structures. In this context, the reduced integrated radius observed in eyes with staphyloma is particularly relevant. The integrated radius represents a function of the inverse radius of curvature throughout the entire corneal movement and is considered a comprehensive indicator of total corneal deflection. Lower values may therefore reflect a reduced capacity to dissipate mechanical energy during the deformation process, potentially allowing for greater transmission of stress toward the posterior pole. The differences observed in several deformation-related parameters, including A1 deformation amplitude, A1 deflection length, and maximum deformation amplitude, may indicate an altered dynamic response to the applied stress; however, this interpretation remains conceptual, given the lack of direct measurements of posterior-segment biomechanics, and should be further tempered by the exploratory nature of the analysis, which involved multiple small subgroup comparisons rather than a single unified confirmatory model.

Within this framework, posterior staphyloma may represent a clinical expression of global ocular biomechanical susceptibility. This interpretation is supported by previous evidence demonstrating increased deformability and altered viscoelastic properties in eyes with staphyloma [34].

Collectively, these findings suggest heterogeneous biomechanical profiles across macular structural phenotypes in highly myopic eyes. Eyes with foveoschisis and posterior staphyloma showed more pronounced differences in corneal dynamic response parameters, whereas eyes with outer retinal atrophy/fibrosis or vitreomacular traction showed less consistent associations. Given the exploratory nature of the analyses and the multiple comparisons performed, these results should be interpreted cautiously and primarily as a basis for future confirmatory studies.

### 4.2. Correlations Between Baseline Biomechanics and Longitudinal Functional Changes

Our analysis further explored associations between baseline ocular biomechanical properties and longitudinal functional changes in highly myopic eyes over a 5-year period. Several baseline Corvis ST parameters were associated with longitudinal changes in fixation stability and retinal sensitivity, suggesting that ocular biomechanical behavior may be associated with longitudinal functional decline.

Among the evaluated outcomes, changes in fixation stability (BCEA indices) showed the most consistent associations with baseline biomechanical parameters. An increase in BCEA over time reflects enlargement of the fixation area and, therefore, deterioration in fixation stability. In our cohort, the correlations observed for BCEA were highly consistent across the three BCEA metrics (1SD, 2SD, and 3SD). As expected, BCEA 1SD, 2SD, and 3SD showed highly concordant correlation patterns due to their intrinsic mathematical relationship. Notably, BCEA worsening was associated with higher SSI values, together with negative correlations with deformation-related parameters such as HC dArc Length, A2 dArc Length, dArcLengthMax, and Max InverseRadius. The observed association between higher SSI values and worsening fixation stability may therefore suggest that global stiffness-related biomechanical properties are associated with longitudinal functional changes in highly myopic eyes. In contrast, several deformation-related parameters demonstrated associations in the opposite direction, indicating that stiffness- and deformation-related metrics may capture different aspects of ocular biomechanical behavior. In addition, a trend toward positive correlations between BCEA worsening and Whole Eye Movement (WEM Max) was observed, although these associations did not reach statistical significance. From a biomechanical perspective, WEM reflects an additional anteroposterior displacement of the globe occurring during the air-puff response and may represent a marker of global ocular movement during corneal deformation. The observed positive correlation between WEM and BCEA worsening may suggest that greater global ocular movement during the air-puff response is associated with reduced fixation stability over time. However, the interpretation of this association remains uncertain, and it should be considered exploratory. Collectively, these findings may suggest that distinct aspects of dynamic corneal response behavior are associated with longitudinal changes in fixation stability.

Changes in CPS also demonstrated several correlations with baseline biomechanical measurements. In contrast to BCEA, a negative change in CPS reflects functional loss. We consider CPS to be more clinically relevant, as it reflects retinal functional performance and may capture subtle functional decline not always evident in structural imaging. In contrast, BCEA is less robust in terms of direct clinical interpretation and should be interpreted with greater caution. CPS decline was associated with several first-generation dynamic corneal response parameters, including A1 time, A2 velocity, and other deformation-related metrics, as well as pressure-related variables. Notably, a longer A1 time was associated with greater CPS reduction, which may indicate a reduced ability of the cornea to rapidly absorb and dissipate the applied energy. In biomechanical terms, delayed first applanation can reflect a less efficient damping response to the air-puff stimulus. In addition, higher biomechanically corrected intraocular pressure (bIOP) and higher SP-A1—reflecting increased resistance to deformation—were both negatively correlated with ΔCPS. These findings may indicate that different components of ocular biomechanical behavior—including deformation velocity, deformation extent, and stiffness-related parameters—are associated with longitudinal changes in retinal sensitivity in highly myopic eyes.

Correlations involving fixation indices F2° and F4° were fewer but showed a pattern generally consistent with the findings observed for BCEA. Specifically, changes in fixation stability were positively associated with A1 Time and negatively associated with A1 Velocity and A2 Deformation Amplitude, suggesting that eyes with slower initial deformation and reduced dynamic response tended to exhibit greater deterioration in fixation stability over time. In addition, positive correlations were observed with HC dArc Length and dArcLengthMax, parameters related to the geometric characteristics of corneal deformation during the air-puff cycle. Although these associations were less numerous than those observed for BCEA, their overall pattern supports the notion that variations in ocular biomechanical behavior may influence the stability of fixation mechanisms in highly myopic eyes.

Multivariable regression analyses identified associations between longitudinal changes in retinal sensitivity (ΔCPS 12°) and selected baseline demographic, anatomical, and biomechanical parameters. After adjustment for age, spherical equivalent, central foveal thickness, and choroidal thickness, A1 Velocity, A2 Velocity, and HC Deflection Length remained associated with ΔCPS 12°. A1 and A2 Velocity describe the speed of corneal movement during the inward and outward phases of deformation, whereas HC Deflection Length reflects the extent of corneal deformation at the highest concavity. Higher deformation velocities were associated with greater declines in retinal sensitivity over time, whereas greater deformation extent was associated with relative functional preservation. Although these associations were observed after adjustment for key anatomical covariates, their interpretation should remain cautious. Given the limited sample size relative to the number of candidate predictors considered and the exploratory nature of the modeling strategy, these multivariable results should not be interpreted as a stable predictive model. Instead, they should be considered hypothesis-generating, warranting validation in larger and independent cohorts. The biological significance of these findings therefore remains uncertain, although the consistency of associations across related dynamic parameters suggests a potential relationship between ocular biomechanical response and functional longitudinal change in highly myopic eyes.

Clinically, corneal and ocular biomechanical assessment may represent a useful adjunct to anatomical imaging and functional testing. Although exploratory and hypothesis-generating, these findings suggest that biomechanical profiling could contribute to future risk stratification approaches and improve understanding of disease heterogeneity in highly myopic eyes. Incorporating biomechanical profiling could therefore improve risk stratification and support more individualized management strategies for highly myopic eyes.

### 4.3. Limitations and Future Perspectives

Several limitations should be considered when interpreting our results. First, the study cohort was relatively small, particularly in the vitreomacular traction subgroup, which may have limited the statistical power to detect subtle biomechanical differences. Second, corneal biomechanical parameters were used as indirect markers of global ocular biomechanical behavior, but no direct assessment of scleral biomechanics was performed. Although parameters such as the stress–strain index and whole-eye-movement metrics have been proposed to reflect aspects of the corneoscleral response beyond the cornea alone, the relationship between corneal dynamic response measurements and posterior scleral biomechanics remains indirect and incompletely understood. Therefore, mechanistic interpretations regarding whole-globe biomechanics or stress transmission to the posterior segment should be considered hypothetical. Third, axial length, a key structural parameter in high myopia, was not available because ocular biometry was not systematically performed in all participants. Consequently, correlations between axial elongation and biomechanical parameters could not be assessed. Finally, the observational nature of the study precludes causal inference.

Given the exploratory nature of this study and the relatively small sample size, these findings should be interpreted cautiously and considered hypothesis-generating. Further larger prospective studies are required to validate these associations and determine their potential clinical applicability.

Despite these limitations, our findings highlight the potential for ocular biomechanics to inform risk stratification and functional prognosis in high myopia. Future research could expand the cohort size, integrate multimodal imaging of the posterior segment, and explore the predictive value of biomechanics for surgical outcomes or disease-modifying interventions. Additionally, longitudinal studies incorporating repeated biomechanical measurements may elucidate how changes in tissue compliance over time relate to macular structural remodeling and functional decline. Importantly, the present interpretation should be regarded as a conceptual and theoretical proposition that remains to be empirically validated in independent cohorts before any clinical translation can be considered. Ultimately, integrating biomechanics with anatomical and functional assessments could enhance personalized management strategies for highly myopic eyes and refine classification systems for pathologic myopia.

## Figures and Tables

**Table 1:** Scheimpflug camera-derived corneal biomechanical parameters with explanation and abbreviations.

Parameter	Abbreviation	Explanation	Anatomical Unit Movement	Theoretical Meaning of Higer Values
cIOP [mmHg]	cIOP	Corvis-derived intraocular pressure	Non applied	Non applied
cPachy [µm]	cCCT	Corvis-derived central corneal thickness	Non applied	Non applied
1st generation parameters				
Deformation Amp. Max [mm]	MaxDefoA	Corneal deformation amplitude during MaxDT, expressed as the sum of corneal deflection amplitude and MaxWEM	Ocular deformation	Less rigid behavior
A1 Time [ms]	A1T	Time from the beginning of measurement to the first applanation moment	Corneal deflection	More rigid behavior
A1 Velocity [m/s]	A1V	Velocity of the corneal apex during the first applanation	Corneal deflection	Less rigid behavior
A2 Time [ms]	A2T	Time from the beginning of measurement to the second applanation moment	Corneal deflection	Less rigid behavior
A2 Velocity [m/s]	A2V	Velocity of the corneal apex during the second applanation	Corneal deflection	Less rigid behavior
HC Time [ms]	HCT	Time from the beginning of measurement to the moment of reaching the highest concavity (HC)	Corneal deflection	Less rigid behavior
Peak Dist. [mm]	HCPD	Distance between the corneal peaks at the HC	Corneal deflection	Less rigid behavior
Radius [mm]	HCR	Radius of corneal curvature during the HC	Corneal deflection	More rigid behavior
A1 Deformation Amp. [mm]	A1DefoA	Corneal deformation amplitude during A1, expressed as the sum of corneal deflection amplitude and MaxWEM	Ocular deformation	Less rigid behavior
HC Deformation Amp. [mm]	HCDefoA	Corneal deformation amplitude during HC, expressed as the sum of corneal deflection amplitude and MaxWEM	Ocular deformation	Less rigid behavior
A2 Deformation Amp. [mm]	A2DefoA	Corneal deformation amplitude during A2, expressed as the sum of corneal deflection amplitude and MaxWEM	Ocular deformation	Less rigid behavior
A1 Deflection Length [mm]	A1DL	Horizontal length of the flattened cornea at the A1	Corneal deflection	More rigid behavior
HC Deflection Length [mm]	HCDL	Horizontal length of the flattened cornea at the HC	Corneal deflection	More rigid behavior
A2 Deflection Length [mm]	A2DL	Horizontal length of the flattened cornea at the A2	Corneal deflection	More rigid behavior
A1 Deflection Amp. [mm]	A1DA	Corneal deflection amplitude during A1, determined as the displacement of the corneal apex in relation to the initial state without MaxWEM quantification	Corneal deflection	Less rigid behavior
HC Deflection Amp. [mm]	HCDA	Corneal deflection amplitude during HC, determined as the displacement of the corneal apex in relation to the initial state without MaxWEM quantification	Corneal deflection	Less rigid behavior
A2 Deflection Amp. [mm]	A2DA	Corneal deflection amplitude during A2, determined as the displacement of the corneal apex in relation to the initial state without MaxWEM quantification	Corneal deflection	Less rigid behavior
Deflection Amp. Max [mm]	MaxDA	Corneal deflection amplitude during MaxDT	Corneal deflection	Less rigid behavior
Deflection Amp. Max [ms]	MaxDT	Moment of the maximum corneal deflection during the oscillatory phase near HC	Corneal deflection	Less rigid behavior
Whole Eye Movement Max [mm]	MaxWEM	Amplitude of the maximum whole eye movement	Ocular deformation	More energy to posterior pole
Whole Eye Movement Max [ms]	MaxWEMT	Time at which the highest amplitude of the maximum whole eye movement (near A2) occurs	Ocular deformation	Less energy to posterior pole
A1 Deflection Area [mm^2^]	A1DArea	Deflection area in A1	Corneal deflection	Less rigid behavior
HC Deflection Area [mm^2^]	HCDArea	Deflection area in HC	Corneal deflection	Less rigid behavior
A2 Deflection Area [mm^2^]	A2DArea	Deflection area in A2	Corneal deflection	Less rigid behavior
A1 dArc Length [mm]	A1dArcL	Delta arc length of the corneal surface in A1	Corneal deflection	Less rigid behavior
HC dArc Length [mm]	HCdArcL	Delta arc length of the corneal surface in HC	Corneal deflection	Less rigid behavior
A2 dArc Length [mm]	A2dArcL	Delta arc length of the corneal surface in A2	Corneal deflection	Less rigid behavior
dArcLengthMax [mm]	MaxdArcL	Delta arc length of the corneal surface in MaxDT	Corneal deflection	Less rigid behavior
2nd generation parameters				
Max Inverse Radius [mm^−1^]	MIR	1/HCR	Corneal deflection	Less rigid behavior
DA Ratio Max (2 mm)	DARM2	Ápex MaxDA/MaxDA at 2 mm from the ápex	Corneal deflection	Less rigid behavior
PachySlope [µm]	PqS	Peripheric (8 mm horizontal) pachymetry/Ápex pachymetry	Non applied	Non applied
DA Ratio Max (1 mm)	DARM1	Ápex MaxDA/MaxDA at 1 mm from the ápex	Corneal deflection	Less rigid behavior
Ambrosio Relational Thickness (8 mm)	ARTh	Ambrosio relational thickness within the horizontal 8 mm cornea of the image	Non applied	Non applied
Biomechanically Corrected IOP	bIOP	IOP adjusted for biomechanical parameters	Non applied	Non applied
Integrated Radius [mm^−1^]	IR	Area under the curve of the 1/HCR function	Corneal deflection	Less rigid behavior
Stiffness parameter in A1	SP-A1	Air-puff pressure–bIOP/A1DA	Ocular deformation	More rigid behavior
Corvis biomechanical index	CBI	Exponential function score determined through a logistic regression analysis of 6 parameters (SP-A1, DARM1, DARM2, ARTh, A1V and MaxDefoA) and adjusted for IOP and CCT to describe ectasia risk	Corneal deflection	Less rigid behavior
Tomographic and Biomechanical Index	TBI	Algorithm including tomographic and biomechanical parameters for the discrimination of eyes with corneal ectasia susceptibility	Corneal deflection	Less rigid behavior
Stress–Strain Index	SS-I	Finite element modeling algorithm for the estimation of the non-linear in vivo biomechanical behavior in corneas with normal topography	Ocular deformation	More rigid behavior

**Table 2:** Overall and per-group analysis of demographic, anatomical, and microperimetric data.

Parameter	All Samples (*N* = 54)	Schisis			Atrophy/Fibrosis			VMT			Posterior Staphyloma		
		Yes (*n* = 19)	No (*n* = 35)	*p*	Yes (*n* = 33)	No (*n* = 21)	*p*	Yes (*n* = 9)	No (*n* = 45)	*p*	Yes (*n* = 31)	No (*n* = 23)	*p*
Age (years)	58.3 ± 12.7	63.0 ± 11.5	55.2 ± 13.1	0.147	60.9 ± 11.7	53.1 ± 13.3	0.134	66.7 ± 10.2	56.2 ± 12.4	0.212	59.2 ± 13.4	56.3 ± 11.7	0.317
Absolute SE (D)	15.5 ± 6.6	14.9 ± 7.6	16.20 ± 5.9	0.608	16.80 ± 7.2	10.8 ± 7.2	0.245	10.4 ± 11.6	10.1 ± 7.9	0.191	10.4 ± 9.5	9.8 ± 7.1	0.269
Anatomical data													
CFT (μm)	216.7 ± 142.1	280.6 ± 194	177.6 ± 80	0.1	192.1 ± 157	264.5 ± 92.9	0.088	329.8 ± 261.2	191.9 ± 87.2	0.447	231.9 ± 169.1	191.9 ± 79.1	0.482
CT (μm)	49.10 ± 55.1	38.30 ± 19.8	67.0 ± 35.0	0.283	32.7 ± 23.9	80.9 ± 80.7	0.027 *	34.1 ± 22.8	52.4 ± 39.6	0.585	49.2 ± 35.3	49.9 ± 43.6	0.486
Microperimetric data													
CPS 12° (dB)	14.37 ± 9.1	13.7 ± 9.1	14.8 ± 9.3	0.973	11.2 ± 8.8	20.9 ± 6.0	<0.001 *	12.8 ± 8.4	14.5 ± 9.4	0.283	12.2 ± 9.1	17.73 ± 8.4	0.394
F2° (%)	78.4 ± 23.5	76.7 ± 26.2	79.5 ± 21.8	0.903	76.6 ± 25.4	81.9 ± 19.2	0.725	62.0 ± 27.9	81.8 ± 21.3	0.332	77.93 ± 23.9	94.1 ± 10.2	0.102
F4° (%)	92.8 ± 13.5	90.5 ± 17.9	94.4 ± 9.3	0.593	91.7 ± 15.4	95.1 ± 8.0	0.591	87.6 ± 12.6	93.9 ± 13.6	0.04 *	92.0 ± 15.3	94.1 ± 10.23	0.466
BCEA 1SD (°)	3.48 ± 5.8	2.2 ± 1.7	2.6 ± 2.8	0.858	4.0 ± 6.8	2.4 ± 2.3	0.842	5.3 ± 4.1	2.7 ± 3.26	0.081	3.0 ± 6.9	7.7 ± 8.7	0.544
BCEA 2SD (°)	9.34 ± 15.6	12.6 ± 11.1	7.1 ± 7.60	0.858	10.8 ± 18.3	6.4 ± 6.1	0.833	14.18 ± 11.1	7.3 ± 8.79	0.368	10.4 ± 18.7	14.7 ± 16.7	0.566
BCEA 3SD (°)	17.88 ± 29.7	11.1 ± 42.8	13.5 ± 14.5	0.526	20.7 ± 35.1	12.1 ± 11.7	0.824	27.1 ± 21.2	12.4 ± 9.37	0.205	19.9 ± 35.8	17.7 ± 8.4	0.439

Note: * Statistical significance for *p* < 0.05. Abbreviations: BCVA, best corrected visual acuity; SE, spherical equivalent; CFT, central 1 mm foveal thickness; CT, subfoveal choroidal thickness; CPS 12, retinal sensitivity in the foveally centered 12° polygon; F2 and F4, proportion of fixations within the 2° and 4° foveally centered circles; BCEA 1SD, 2SD and 3SD, bivariate contour ellipse areas of the 1 (68.2%), 2 (95.4%) and 3 (99.6%) standard deviations of the total fixations.

**Table 3:** (**a**) Biomechanical differences between eyes with different myopia-related pathological changes (*n* = 54) (foveoschisis and atrophy/fibrosis groups). (**b**) Biomechanical differences between eyes with different myopia-related pathological changes (*n* = 54) (vitreomacular and staphyloma groups).

**(a)**										
	**Foveoschisis**					**Atrophy/Fibrosis**				
	**Yes (*****n*** **= 19)**		**No (*****n*** **= 35)**		***p***	**Yes (*****n*** **= 33)**		**No (*****n*** **= 21)**		***p***
	**Mean**	**SD**	**Mean**	**SD**		**Mean**	**SD**	**Mean**	**SD**	
Pachy [µm]	519.000	39.569	535.179	37.221	0.191	529.200	32.835	530.308	50.501	0.932
Def, Amp, Max [mm]	1.175	0.130	1.171	0.181	0.935	1.190	0.176	1.132	0.126	0.290
A1 Time [ms]	7.923	0.413	7.967	0.452	0.754	7.922	0.424	8.021	0.467	0.497
A1 Velocity [m/s]	0.134	0.019	0.128	0.027	0.450	0.134	0.019	0.122	0.033	0.113
A2 Time [ms]	21.781	0.383	21.768	0.571	0.936	21.792	0.472	21.729	0.595	0.713
A2 Velocity [m/s]	−0.301	0.042	−0.311	0.047	0.486	−0.310	0.047	−0.302	0.040	0.576
HC Time [ms]	17.278	0.478	17.224	0.737	0.799	17.132	0.723	17.498	0.354	0.032 *
Peak Dist, [mm]	5.322	0.290	5.341	0.411	0.881	5.372	0.386	5.246	0.325	0.310
Radius [mm]	6.990	1.046	6.686	0.954	0.341	6.861	0.937	6.633	1.113	0.493
A1 Deformation Amp, [mm]	0.145	0.013	0.143	0.021	0.678	0.146	0.018	0.137	0.017	0.136
HC Deformation Amp, [mm]	1.175	0.130	1.171	0.181	0.935	1.190	0.176	1.132	0.126	0.290
A2 Deformation Amp, [mm]	0.371	0.049	0.333	0.044	0.014 *	0.350	0.048	0.341	0.052	0.618
A1 Deflection Length [mm]	2.317	0.217	2.327	0.172	0.876	2.336	0.169	2.289	0.235	0.489
HC Deflection Length [mm]	6.937	0.558	6.797	0.625	0.475	6.915	0.616	6.677	0.541	0.249
A2 Deflection Length [mm]	3.392	1.076	2.631	0.626	0.007 *	2.974	0.947	2.753	0.737	0.474
A1 Deflection Amp, [mm]	0.094	0.011	0.109	0.107	0.598	0.094	0.008	0.126	0.059	0.487
HC Deflection Amp, [mm]	1.034	0.144	1.051	0.220	0.783	1.049	0.187	1.037	0.220	0.854
A2 Deflection Amp, [mm]	0.115	0.017	0.128	0.099	0.634	0.109	0.014	0.155	0.140	0.265
Deflection Amp, Max [mm]	1.050	0.140	1.104	0.240	0.358	1.083	0.202	1.089	0.238	0.934
Deflection Amp, Max [ms]	16.143	0.518	16.087	0.933	0.802	16.178	0.768	15.940	0.899	0.381
Whole Eye Movement Max [mm]	0.262	0.052	0.239	0.076	0.306	0.255	0.065	0.230	0.078	0.277
Whole Eye Movement Max [ms]	22.102	0.571	21.259	4.199	0.446	22.092	0.901	20.311	1.022	0.309
A1 Deflection Area [mm^2^]	0.182	0.027	0.204	0.236	0.724	0.177	0.028	0.240	0.348	0.522
HC Deflection Area [mm^2^]	3.958	0.719	3.943	1.020	0.961	4.061	0.977	3.689	0.732	0.226
A2 Deflection Area [mm^2^]	0.276	0.069	0.294	0.267	0.801	0.249	0.060	0.374	0.374	0.250
A1 DArc Length [mm]	−0.019	0.005	−0.020	0.029	0.838	−0.018	0.003	−0.022	0.043	0.758
HC DArc Length [mm]	−0.149	0.030	−0.113	0.104	0.206	−0.144	0.034	−0.082	0.144	0.151
A2 DArc Length [mm]	−0.025	0.009	−0.028	0.022	0.658	−0.024	0.006	−0.033	0.031	0.303
DArcLengthMax [mm]	−0.168	0.026	−0.170	0.051	0.880	−0.167	0.043	−0.174	0.048	0.648
Max Inverse Radius [mm^−1^]	0.182	0.025	0.211	0.087	0.206	0.189	0.041	0.229	0.114	0.244
DA Ratio Max (2 mm)	4.014	0.364	4.609	2.677	0.399	4.060	0.555	5.189	3.855	0.314
PachySlope [µm]	43.190	24.215	37.891	27.645	0.536	34.890	19.260	50.930	36.547	0.156
DA Ratio Max (1 mm)	1.516	0.053	1.571	0.244	0.397	1.517	0.057	1.632	0.350	0.260
ARTh	488.963	265.053	629.380	369.792	0.201	581.876	318.455	576.986	400.736	0.969
bIOP	15.253	3.013	15.562	2.971	0.752	15.132	3.021	16.131	2.788	0.320
Integrated Radius [mm^−1^]	8.418	1.663	8.901	1.699	0.376	8.625	1.579	8.980	1.947	0.531
SP A1	115.040	24.809	110.507	32.812	0.647	115.519	23.875	104.479	40.715	0.289
CBI	0.380	0.333	0.422	0.399	0.741	0.365	0.323	0.506	0.469	0.377
TBI	6.273	0.670	6.321	0.850	0.857	6.343	0.670	6.189	1.033	0.669
SSI	0.986	0.281	0.827	0.196	0.035 *	0.905	0.258	0.830	0.184	0.289
**(b)**										
	**Vitreomacular Traction**					**Staphyloma**				
	**Yes (*****n*** **= 9)**		**No (*****n*** **= 45)**		***p***	**Yes (*****n*** **= 31)**		**No (*****n*** **= 23)**		***p***
	**Mean**	**SD**	**Mean**	**SD**		**Mean**	**SD**	**Mean**	**SD**	
Pachy [µm]	532.750	32.539	528.800	39.989	0.796	537.536	38.373	514.600	34.834	0.061
Def, Amp, Max [mm]	1.174	0.141	1.172	0.170	0.97	1.146	0.160	1.220	0.164	0.163
A1 Time [ms]	8.042	0.496	7.931	0.424	0.519	8.032	0.474	7.802	0.308	0.098
A1 Velocity [m/s]	0.128	0.029	0.131	0.024	0.787	0.132	0.021	0.128	0.030	0.608
A2 Time [ms]	21.678	0.425	21.795	0.527	0.560	21.670	0.488	21.958	0.502	0.076
A2 Velocity [m/s]	−0.298	0.055	−0.310	0.043	0.516	−0.306	0.045	−0.311	0.046	0.752
HC Time [ms]	17.389	0.607	17.210	0.666	0.489	17.093	0.634	17.523	0.611	0.038
Peak Dist, [mm]	5.334	0.371	5.334	0.375	0.998	5.293	0.388	5.410	0.333	0.329
Radius [mm]	6.889	1.243	6.770	0.937	0.763	7.126	0.896	6.168	0.850	0.002 *
A1 Deformation Amp, [mm]	0.145	0.014	0.143	0.019	0.802	0.150	0.019	0.132	0.008	0.001 *
HC Deformation Amp, [mm]	1.174	0.141	1.172	0.170	0.970	1.146	0.160	1.220	0.164	0.163
A2 Deformation Amp, [mm]	0.372	0.045	0.341	0.049	0.105	0.341	0.051	0.358	0.043	0.261
A1 Deflection Length [mm]	2.298	0.219	2.329	0.183	0.694	2.369	0.178	2.225	0.173	0.019 *
HC Deflection Length [mm]	6.837	0.673	6.849	0.594	0.963	6.873	0.571	6.796	0.671	0.698
A2 Deflection Length [mm]	3.350	1.453	2.819	0.722	0.151	2.920	0.943	2.890	0.802	0.921
A1 Deflection Amp, [mm]	0.165	0.190	0.090	0.027	0.302	0.097	0.009	0.116	0.148	0.618
HC Deflection Amp, [mm]	1.098	0.271	1.033	0.176	0.406	1.013	0.180	1.105	0.215	0.144
A2 Deflection Amp, [mm]	0.175	0.175	0.111	0.023	0.341	0.113	0.016	0.143	0.132	0.392
Deflection Amp, Max [mm]	1.116	0.267	1.078	0.199	0.650	1.028	0.175	1.192	0.235	0.013 *
Deflection Amp, Max [ms]	15.722	0.760	16.194	0.801	0.137	16.044	0.681	16.223	1.016	0.494
Whole Eye Movement Max [mm]	0.246	0.087	0.248	0.066	0.950	0.243	0.069	0.255	0.070	0.580
Whole Eye Movement Max [ms]	19.649	0.783	21.989	0.906	0.424	21.984	0.837	20.751	0.694	0.418
A1 Deflection Area [mm^2^]	0.330	0.019	0.165	0.056	0.305	0.183	0.027	0.220	0.325	0.666
HC Deflection Area [mm^2^]	3.901	0.859	3.959	0.942	0.874	3.899	0.966	4.041	0.843	0.635
A2 Deflection Area [mm^2^]	0.311	0.205	0.282	0.222	0.736	0.254	0.064	0.348	0.352	0.319
A1 DArc Length [mm]	−0.035	0.046	−0.016	0.013	0.295	−0.019	0.004	−0.020	0.040	0.922
HC DArc Length [mm]	−0.108	0.122	−0.130	0.079	0.534	−0.152	0.031	−0.076	0.130	0.041 *
A2 DArc Length [mm]	−0.030	0.021	−0.026	0.018	0.558	−0.025	0.007	−0.030	0.029	0.530
DArcLengthMax [mm]	−0.189	0.057	−0.165	0.040	0.168	−0.169	0.041	−0.169	0.051	0.999
Max InverseRadius [mm^−1^]	0.208	0.099	0.199	0.067	0.772	0.175	0.020	0.249	0.105	0.016 *
DA Ratio Max (2 mm)	5.800	4.867	4.082	0.635	0.352	3.912	0.477	5.316	3.519	0.146
PachySlope [µm]	38.140	12.900	40.105	28.661	0.852	34.154	23.374	50.164	29.111	0.079
DA Ratio Max (1 mm)	1.575	0.108	1.547	0.217	0.729	1.502	0.059	1.646	0.315	0.101
ARTh	443.206	202.008	611.755	359.642	0.088	614.132	348.878	517.425	326.301	0.381
bIOP	15.638	3.684	15.403	2.813	0.843	15.842	3.425	14.767	1.782	0.194
Integrated Radius [mm^−1^]	8.218	1.261	8.850	1.759	0.344	8.164	1.428	9.794	1.646	0.002 *
SP A1	105.129	45.899	113.976	25.017	0.460	118.152	26.701	101.165	33.050	0.086
CBI	0.399	0.339	0.407	0.383	0.957	0.351	0.312	0.498	0.451	0.294
TBI	6.319	0.410	6.297	0.840	0.948	6.408	0.753	6.104	0.797	0.257
SSI	0.949	0.190	0.867	0.248	0.389	0.917	0.245	0.818	0.220	0.202

Note: (**a**) * Statistical significance for *p* < 0.05. Comparisons between groups were performed using the Mann–Whitney test for all variables except Pachymetry (Pachy [µm]), Deflection Amplitude Maximum (Def, Amp, Max [mm]), Pachymetry Slope (PachySlope [µm]), and Stress–Strain Index (SSI). Abbreviations: cIOP, corrected intraocular pressure; Pachy, central corneal thickness; Def. Amp. Max, maximum deformation amplitude; A1 and A2, first and second applanation events; HC, highest concavity; Time, time to event; Velocity, corneal velocity at applanation; Peak Dist., peak distance; Radius, radius of curvature at highest concavity; Deflection Length, corneal deflection length; Deflection Amp., corneal deflection amplitude; Deflection Area, area of corneal deflection; dArc Length, differential arc length; dArcLengthMax, maximum differential arc length; Max InverseRadius, maximum inverse radius of curvature; DA Ratio Max (1 mm and 2 mm), maximum deformation amplitude ratio at 1 mm and 2 mm from the apex; PachySlope, corneal pachymetric progression slope; ARTh, Ambrósio relational thickness; bIOP, biomechanically corrected intraocular pressure; Integrated Radius, integrated inverse radius; SP A1, stiffness parameter at first applanation; CBI, Corvis biomechanical index; TBI, tomographic biomechanical index; SSI, stress–strain index. (**b**) * Statistical significance for *p* < 0.05. Comparisons between groups were performed using the Mann–Whitney test for all variables except Pachymetry (Pachy), Deflection Amplitude Maximum (Def, Amp, Max), Pachymetry Slope (PachySlope), and Stress–Strain Index (SSI). Abbreviations: cIOP, corrected intraocular pressure; Pachy, central corneal thickness; Def. Amp. Max, maximum deformation amplitude; A1 and A2, first and second applanation events; HC, highest concavity; Time, time to event; Velocity, corneal velocity at applanation; Peak Dist., peak distance; Radius, radius of curvature at highest concavity; Deflection Length, corneal deflection length; Deflection Amp., corneal deflection amplitude; Deflection Area, area of corneal deflection; dArc Length, differential arc length; dArcLengthMax, maximum differential arc length; Max InverseRadius, maximum inverse radius of curvature; DA Ratio Max (1 mm and 2 mm), maximum deformation amplitude ratio at 1 mm and 2 mm from the apex; PachySlope, corneal pachymetric progression slope; ARTh, Ambrósio relational thickness; bIOP, biomechanically corrected intraocular pressure; Integrated Radius, integrated inverse radius; SP A1, stiffness parameter at first applanation; CBI, Corvis biomechanical index; TBI, tomographic biomechanical index; SSI, stress–strain index.

**Table 4:** Longitudinal changes in macular microperimetric functional outcomes and correlation analyses between them and baseline ocular biomechanical parameters (*n* = 54).

	Δ_F2°		Δ_F4°		Δ_BCEA_1SD		Δ_BCEA_2SD		Δ_BCEA_3SD		Δ_CPS_12°	
	Mean	SD	Mean	SD	Mean	SD	Mean	SD	Mean	SD	Mean	SD
	−15.0	38.319	−10.03	12.493	4.480	6.719	12.089	18.056	23.143	34.568	−3.559	6.417
	Pearson Correlations											
	*r*	*p*	*r*	*p*	*r*	*p*	*r*	*p*	*r*	*p*	*r*	*p*
IOP [mmHg]	0.347	0.060 *	0.178	0.338	−0.116	0.534	−0.116	0.534	−0.116	0.534	−0.457	0.011 *
Pachy [µm]	0.162	0.391	0.056	0.764	−0.005	0.978	−0.004	0.982	−0.005	0.978	−0.175	0.356
Def. Amp. Max [mm]	−0.298	0.110	−0.219	0.237	0.121	0.518	0.120	0.519	0.120	0.519	0.379	0.039 *
A1 Time [ms]	0.338	0.068 *	0.196	0.291	−0.122	0.512	−0.122	0.513	−0.122	0.512	−0.453	0.012 *
A1 Velocity [m/s]	−0.385 *	0.036 *	−0.266	0.148	0.175	0.346	0.175	0.347	0.175	0.346	0.359	0.051 *
A2 Time [ms]	−0.277	0.138	−0.106	0.577	0.040	0.835	0.041	0.832	0.041	0.832	0.456	0.013 *
A2 Velocity [m/s]	−0.157	0.407	−0.139	0.465	0.121	0.524	0.122	0.520	0.122	0.520	−0.529	0.003 *
HC Time [ms]	−0.246	0.189	−0.006	0.973	0.033	0.860	0.032	0.863	0.033	0.862	−0.010	0.960
Peak Dist. [mm]	−0.151	0.424	−0.142	0.446	0.012	0.948	0.012	0.948	0.012	0.949	0.404	0.027 *
Radius [mm]	0.039	0.836	−0.202	0.276	0.283	0.123	0.283	0.123	0.283	0.122	−0.245	0.191
A1 Deformation Amp. [mm]	0.154	0.417	−0.138	0.458	0.295	0.107	0.296	0.106	0.296	0.106	−0.128	0.500
HC Deformation Amp. [mm]	−0.298	0.110	−0.219	0.237	0.121	0.518	0.120	0.519	0.120	0.519	0.379	0.039 *
A2 Deformation Amp. [mm]	−0.369 *	0.045 *	−0.242	0.197	0.327	0.078	0.327	0.078	0.328	0.077	−0.038	0.844
A1 Deflection Length [mm]	−0.431 *	0.017 *	0.261	0.156	−0.066	0.725	−0.066	0.725	−0.065	0.728	0.078	0.681
HC Deflection Length [mm]	−0.036	0.848	−0.163	0.382	0.113	0.544	0.113	0.545	0.113	0.544	0.482	0.007 *
A2 Deflection Length [mm]	−0.125	0.511	−0.384	0.036 *	0.375	0.041 *	0.374	0.042 *	0.374	0.042 *	0.081	0.675
A1 Deflection Amp. [mm]	−0.129	0.496	−0.018	0.924	0.215	0.245	0.215	0.246	0.215	0.244	−0.041	0.832
HC Deflection Amp. [mm]	−0.257	0.170	−0.146	0.433	0.021	0.909	0.021	0.910	0.021	0.910	0.423	0.020 *
A2 Deflection Amp. [mm]	−0.091	0.632	−0.061	0.750	0.324	0.080	0.323	0.082	0.324	0.081	0.209	0.276
Deflection Amp. Max [mm]	−0.267	0.154	−0.160	0.391	0.040	0.829	0.040	0.830	0.040	0.830	0.424	0.019 *
Deflection Amp. Max [ms]	−0.134	0.479	−0.127	0.495	0.121	0.517	0.121	0.517	0.121	0.517	−0.004	0.984
Whole Eye Movement Max [mm]	−0.369 *	0.045 *	−0.256	0.165	0.325	0.074 *	0.326	0.073	0.326	0.073	−0.125	0.511
Whole Eye Movement Max [ms]	−0.318	0.087 *	−0.071	0.704	0.095	0.612	0.096	0.609	0.096	0.608	0.062	0.744
A1 Deflection Area [mm^2^]	0.354	0.055 *	0.156	0.403	−0.013	0.945	−0.013	0.946	−0.012	0.949	−0.047	0.806
HC Deflection Area [mm^2^]	−0.213	0.259	−0.157	0.399	0.037	0.844	0.037	0.844	0.037	0.844	0.434	0.017 *
A2 Deflection Area [mm^2^]	0.272	0.146	−0.028	0.885	0.222	0.238	0.221	0.241	0.222	0.238	0.312	0.099 *
A1 dArc Length [mm]	0.073	0.702	−0.120	0.521	−0.048	0.800	−0.046	0.805	−0.047	0.802	0.084	0.658
HC dArc Length [mm]	0.188	0.321	0.385	0.032 *	−0.380	0.035 *	−0.380	0.035 *	−0.380	0.035 *	−0.443	0.014 *
A2 dArc Length [mm]	−0.010	0.958	0.178	0.348	−0.368	0.045 *	−0.367	0.046 *	−0.368	0.045 *	−0.172	0.371
dArcLengthMax [mm]	0.284	0.129	0.429	0.016 *	−0.406	0.023 *	−0.406	0.023 *	−0.406	0.023 *	−0.441	0.015 *
Max InverseRadius [mm^−1^]	−0.116	0.543	0.277	0.131	−0.367	0.042 *	−0.368	0.042 *	−0.368	0.042 *	0.188	0.321
DA Ratio Max (2 mm)	−0.356	0.053	−0.092	0.621	−0.110	0.555	−0.110	0.555	−0.110	0.556	0.128	0.502
PachySlope [µm]	0.084	0.658	0.169	0.364	−0.255	0.165	−0.255	0.166	−0.255	0.166	0.249	0.185
DA Ratio Max (1 mm)	−0.489	0.006 *	−0.274	0.135	0.043	0.820	0.043	0.820	0.043	0.820	0.021	0.912
ARTh	0.304	0.102	0.268	0.146	−0.287	0.118	−0.287	0.117	−0.288	0.116	−0.225	0.232
bIOP	0.399	0.029 *	0.228	0.217	−0.203	0.273	−0.204	0.272	−0.204	0.272	−0.501	0.005 *
Integrated Radius [mm^−1^]	−0.197	0.298	0.051	0.784	−0.090	0.629	−0.090	0.630	−0.090	0.630	0.282	0.131
SP A1	0.224	0.235	0.038	0.837	−0.030	0.872	−0.030	0.873	−0.031	0.870	−0.377	0.040 *
CBI	−0.147	0.456	0.056	0.773	−0.066	0.733	−0.066	0.734	−0.066	0.735	0.331	0.085
TBI	0.133	0.499	0.022	0.908	−0.025	0.898	−0.025	0.896	−0.026	0.894	−0.178	0.365
SSI	−0.086	0.651	−0.210	0.256	0.411	0.022 *	0.411	0.021 *	0.412	0.021 *	−0.079	0.678

Note: * Statistical significance for *p* < 0.05. Abbreviations: cIOP, corrected intraocular pressure; Pachy, central corneal thickness; Def. Amp. Max, maximum deformation amplitude; A1 and A2, first and second applanation events; HC, highest concavity; Time, time to event; Velocity, corneal velocity at applanation; Peak Dist., peak distance; Radius, radius of curvature at highest concavity; Deflection Length, corneal deflection length; Deflection Amp., corneal deflection amplitude; Deflection Area, area of corneal deflection; dArc Length, differential arc length; dArcLengthMax, maximum differential arc length; Max InverseRadius, maximum inverse radius of curvature; DA Ratio Max (1 mm and 2 mm), maximum deformation amplitude ratio at 1 mm and 2 mm from the apex; PachySlope, corneal pachymetric progression slope; ARTh, Ambrósio relational thickness; bIOP, biomechanically corrected intraocular pressure; Integrated Radius, integrated inverse radius; SP A1, stiffness parameter at first applanation; CBI, Corvis biomechanical index; TBI, tomographic biomechanical index; SSI, stress–strain index; CPS 12, retinal sensitivity in the foveal centered 12° polygon; F2 and F4, proportion of fixations within the 2° and 4° foveal centered circles; BCEA 1SD, 2SD and 3SD, bivariate contour ellipse areas of the 1 (68.2%), 2 (95.4%) and 3 (99.6%) standard deviations of the total fixations; SD, standard deviation.

**Table 5:** Final multivariable linear regression model evaluating associations between baseline Corvis ST biomechanical parameters and longitudinal changes in retinal sensitivity (ΔCPS 12°) over the 5-year follow-up period. The model was adjusted for age, spherical equivalent (SE), central foveal thickness (CFT), and choroidal thickness (CT).

Variable	β Coefficient	*p*-Value
Age	0.284	0.007 *
SE	0.223	0.047 *
Central Foveal Thickness	−0.011	0.056
Choroidal Thickness	0.070	0.001 *
A2 Velocity [m/s]	−85.29	0.003 *
A1 Velocity [m/s]	−239.98	0.004 *
HC Deflection Length [mm]	5.80	0.029 *

Note: * Statistical significance for *p* < 0.05.

## Data Availability

Renato Ambrosio Junior is a consultant for Oculus ^®^.

## Abbreviations

Biomechanical parameters, as well as their abbreviations and definitions, are detailed in Table 1. The following abbreviations are used in this manuscript:

BCEA	Bivariate Contour Ellipse Area
CFT	Central foveal thickness
CPS	Central point sensitivity
CT	Choroidal thickness
F2	Fixation within 2 central degrees
F4	Fixation within 4 central degrees
FS	Foveoschisis
HM	High myopia
IOP	Intraocular pressure
MH	Macular hole
MP-3	Microperimeter MP-3
MTM	Myopic traction maculopathy
OCT	Optical coherence tomography
PM	Pathologic myopia
PS	Posterior staphyloma
SD	Standard deviation
SD-OCT	Spectral-domain optical coherence tomography
SE	Spherical equivalent
VMT	Vitreomacular traction
WEM	Whole eye movement
